# Effect of intravenous dexamethasone on the duration of hyperbaric bupivacaine spinal anesthesia in lower abdominal surgery, Randomized controlled trial

**DOI:** 10.1186/s12871-023-02282-y

**Published:** 2023-09-22

**Authors:** Amani H. Abdel-Wahab, Essam S. Abd Alla, Taghreed Abd El-Azeem

**Affiliations:** Anesthesia and Intensive Care Department, Faculty of Medicine, Assiut University, Assiut, Egypt

**Keywords:** Intravenous, Dexamethasone, Spinal anesthesia, Bupivacaine, Sensory block, Postoperative analgesia

## Abstract

**Background:**

The purpose of this study was to investigate the effect of intravenous (IV) dexamethasone on the duration of hyperbaric bupivacaine spinal anesthesia.

**Methods:**

Two hundred patients between the ages of 18 and 60, of both sexes with ASA I- II classification scheduled for lower abdominal surgery under spinal anesthesia using hyperbaric bupivacaine 0.5% were randomly divided into two groups: the dexamethasone group (Dexa group) and the control group, with 100 patients in each group. Before the administration of spinal anesthesia, the Dexa group received an intravenous infusion of 8 mg dexamethasone in 500 mL normal saline, while the control group received 500 mL normal saline only. The primary outcome of this study was to assess the effect of IV dexamethasone on the regression of hyperbaric bupivacaine spinal anesthesia. Secondary outcome measures included the total duration of sensory and motor blocks, VAS score, time of first analgesic request, total analgesic consumption within the first 24 h, and the occurrence of any side effects.

**Results:**

The Dexa group had significantly delayed onset of 2 dermatomes regression (P < 0.001) compared to the control group. Additionally, the Dexa group had significantly longer duration of both sensory block (P = 0.01) and motor block (P < 0.001). The Dexa group had significantly longer duration until the first postoperative analgesic request (P < 0.001) and a lower incidence of side effects compared to the control group.

**Conclusion:**

Although the intravenous administration of dexamethasone had a limited effect on the duration of hyperbaric bupivacaine spinal anesthesia, it improved postoperative VAS scores compared to the control group and decreased overall postoperative analgesic consumption. Therefore, it can be considered a valuable addition to postoperative multimodal analgesia strategies, aiming to minimize total analgesic consumption.

**Clinical trial registration:**

ID: NCT04778189 (2/3/2021).

## Introduction

Spinal anesthesia is widely used in lower abdominal surgeries, as it plays a crucial role in relieving postoperative pain and enabling ambulatory anesthesia [1]. However, the effect of the block tends to be relatively short, prompting the use of various adjuvants to prolong the duration of the sensory block [2]. However, it is important to note that each adjuvant carries its own set of advantages and disadvantages [3].

Dexamethasone is commonly used to reduce the occurrence of postoperative nausea and vomiting [4]. Additionally, it has been shown to decrease the requirement for postoperative opioids and shorten hospital stays without any reported side effects [5, 6].

Multiple studies have reported that administration of dexamethasone, whether perineurally or intravenously, can prolong the duration of peripheral nerve blocks. However, there is still uncertainty regarding which route is more effective [7]. It is important to note that the perineural route is still considered off-label [6], and there is an ongoing debate about whether the analgesic effect of perineural dexamethasone is due to its systemic effects [8]. Systemic administration of dexamethasone has been found to possess anti-inflammatory and immunosuppressive properties, which may contribute to the prolonged analgesia when administered intravenously [9]. Based on these findings, the intravenous route could be considered as an alternative to the perineural route (9).

Therefore, we conducted this study to examine the effect of intravenous (IV) dexamethasone on the duration of hyperbaric bupivacaine spinal anesthesia in patients undergoing lower abdominal surgery.

## Patients and methods

The study was conducted at the Department of General Surgery, Assiut University main hospital, in accordance with the declarations of Helsinki. Approval was obtained from the local ethics committee with the IRB no:17101550, and the study was registered as a clinical trial with the ID: NCT04778189. Written informed consent was obtained from all patients who participated in this monocentric double-blind randomized controlled study. A total of 200 patients, both male and female, aged between 18 and 60 years old, and classified as American Society of Anesthesiologists (ASA) physical status I- II scheduled for lower abdominal surgery under spinal anesthesia were randomly divided into two groups, the dexamethasone group (Dexa group) and the control group, with 100 patients in each group. Patients with a history of allergy to amide local anesthetics (LAs) or dexamethasone, preexisting lower limb neurological deficit, or chronic use of corticosteroids were excluded from the study.

### Randomization and blinding

Randomization of patients was performed by an independent researcher using computer-generated random tables (http://www.random.org/). The intravenous solutions for the study were prepared by an independent researcher and placed in coded envelopes according to the randomization order. The attending anesthesiologist then opened the envelopes just before the infusion began.

During the preoperative visit, we collected demographic data from all patients. Additionally, we provided training on how to evaluate their postoperative pain using the Visual Analogue Scale (VAS) score. The VAS involves using a ruler numbered from 0 to 10, where 0 represents no pain, 1–3 indicates mild pain, 4–6 represents moderate pain, and 7–10 indicates severe pain [10].

In the operating room, the standard monitors were placed and an 18 gauge peripheral IV cannula was inserted. The Dexa group received an IV infusion of 8 mg dexamethasone in 500 mL normal saline, while the control group received 500 mL normal saline only. After this, an aseptic technique was used to perform an intrathecal injection of 20 mg bupivacaine 0.5% by inserting a 25-G pencil-point Pencan (B. Braun, Mississauga, Ontario, Canada) needle intrathecally at the L4-5 or L3-4 interspace, with the patient in a seated position. The correct intrathecal positioning was confirmed by observing the flow of cerebrospinal fluid through the needle. After completing the intrathecal injection, the patient was turned to a supine position. Then, the sensory level (determined by the absence of sensation to pinprick) and motor level (evaluated using the modified Bromage score) [11] were assessed every two minutes. The surgery began once a satisfactory spinal block level (T7) was achieved. Following the completion of the surgery, patients were transferred to the post-anesthesia care unit (PACU).

The primary outcome measure of this study was to assess the impact of intravenous administration of 8 mg dexamethasone compared to placebo on the regression of hyperbaric bupivacaine spinal anesthesia. The secondary outcome measures included evaluating the total duration of sensory block (time from the highest sensory level to the time of sensory regression to L_1_ or the onset of pain at the surgical site), total duration of motor block (time from modified Bromage score 3 to modified Bromage score 0), Visual Analogue Scale (VAS) score, time of the first analgesic request, total analgesic consumption within the first 24 h, and the occurrence of side effects such as hypotension, bradycardia, nausea, vomiting, and headache.

### Data collection

The attending anesthesiologist evaluated the level of sensory and motor blockade at 5, 10, 20, 30,35, and 40 min after the injection of LA. Subsequently, evaluations were conducted every 15 min until there was regression of 2 dermatomes from the highest level. After the surgery, patients were transferred to the (PACU). In the PACU, the following observations were recorded: the total duration of sensory block, the total duration of motor block, VAS score for evaluating acute postoperative pain, the time of the first postoperative analgesic request (patients received 30 mg ketorolac if their VAS scores were ≥ 3), the total analgesic requirement in the first 24 h, and the occurrence of any side effects such as hypotension, bradycardia, nausea, vomiting, and headache. These observations were performed by data collecting personnel who were blinded to the group assignment, as were the attending anesthesiologist, surgeon, and patient.

### Sample size calculation

The primary outcome of this study was time of 2 dermatomes regression of sensory level in relation to the maximal level. The sample size was calculated using G*power, version 3.1.9.2. Based on a previous study [12] the effect size (d) was determined to be 0.4 after converting the median and interquartile range to mean and standard deviation [13], the effect size d = 0.4. With a power of 80% (using a two-sided t-test and an α level of 0.5) the estimated sample size needed for the study was approximately 200 patients (100 in each group).

### Statistical analysis

We used SPSS version 22. Data were presented as mean (SD), number, and percentage. Parametric and nonparametric tests were applied according to the data distribution. In the case of parametric data, an independent samples t-test was used to compare quantitative variables between the two groups. While in the case of nonparametric data, the Mann-Whitney test was used to compare quantitative variables between the two groups, Chi-square and Fisher Exact tests were used to compare qualitative variable. The P-value was considered statistically significant when P < 0.05.

## Results

**Fig. 1:**
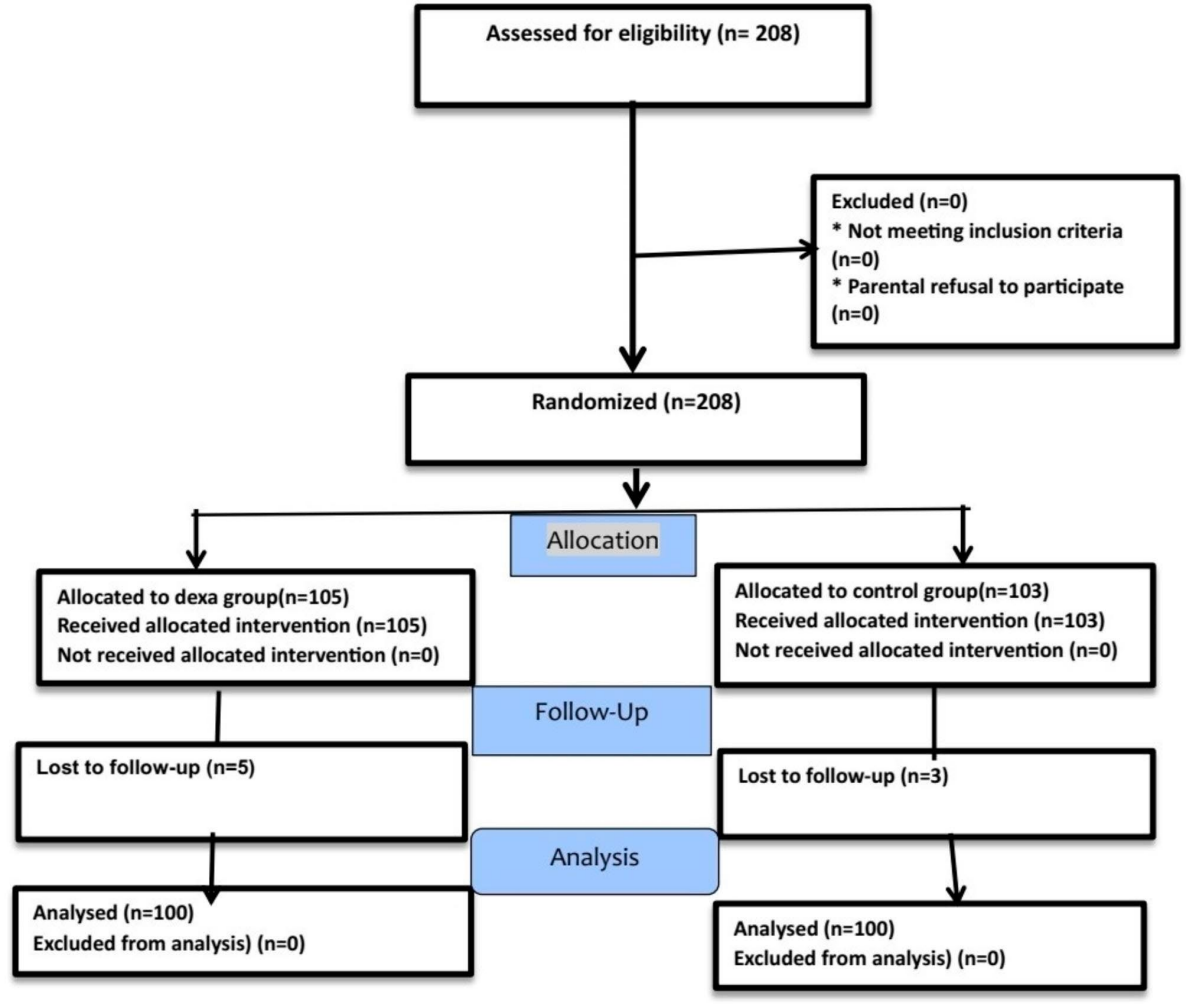
Participant flow diagram

A total of 208 patients were initially considered for this study. However, eight patients were excluded due to a failure of follow-up, resulting in a final sample size of 200 patients who were included in the analysis. (Fig. 1)

Both groups showed no differences in baseline data including age, body mass index, ASA classification, type of surgery, and operative time. (Table 1)

**Table 1:** Baseline data of the studied groups

	Dexa group (n = 100)	Control group (n = 100)	*P* value*
Age (years)	42.28 ± 13.93	42.59 ± 14.30	0.87
Sex			0.37
Male Female	72 (72%) 28 (28%)	75 (75%) 25 (25%)	
BMI (kg/m^2^)	27.21 ± 2.43	28.21 ± 2.03	0.22
Diabetes mellitus	2 (2%)	6 (6%)	0.14
Hypertension	13 (13%)	11 (11%)	0.41
ASA			0.56
Class-I Class-II	85 (85%) 15 (15%)	84 (84%) 16 (16%)	
Surgeries			
Abdominal hernia	57 (57%)	56 (56%)	0.30
Testicular variceal ligation	16 (16%)	18 (18%)	
Hydrocelectomy	19 (19%)	14 (14%)	
Colostomy closure	2 (2%)	5 (5%)	
Fracture penis	1 (1%)	4 (4%)	
Testicular torsion	2 (2%)	3 (3%)	
Hydrocele	3 (3%)	0	
Operative time (minute)	91.51 ± 2.55	90.34 ± 3.89	0.17

Data expressed as frequency (percentage), mean (SD). *P* value was significant if < 0.05. BMI: body mass index; ASA: American Society of Anesthesiologists

*Data were compared by Chi^2^ test (nominal data) and Student t test (continuous data)

Both groups did not show any significant differences as regard the onset of maximum sensory level (32.60 ± 2.79 vs. 32.70 ± 2.51 min; P = 0.79). However, the Dexa group demonstrated a significantly delayed onset of 2 dermatomes regression (91.45 ± 7.45 vs. 87.85 ± 5.91 min; P < 0.001) compared to the control group. Additionally, the Dexa group had a significantly longer duration of sensory block (130.56 ± 26.87 vs. 98.87 ± 13.34 min; P = 0.01) and motor block (230.88 ± 34.87 vs. 165.76 ± 34.43 min; P < 0.001). (Table 2)

**Table 2:** Sensory block, motor block, analgesia, and VAS among the studied groups

	Dexa group (n = 100)	Control group (n = 100)	*P* value*
Onset to maximum sensory level (minute)	32.60 ± 2.79	32.70 ± 2.51	0.79
Onset to regression of 2nd dermatome (minute)	91.45 ± 7.45	87.85 ± 5.91	< 0.001
Duration of sensory block(minute)	130.56 ± 26.87	98.87 ± 13.34	0.01
Duration of motor block (minute)	230.88 ± 34.87	165.76 ± 34.43	< 0.001
Time to 1st analgesia (hour)	4.65 ± 1.58	3.80 ± 1.52	< 0.001
Total analgesic in 1st 24 h (mg)	44.40 ± 22.75	57.86 ± 18.90	< 0.001
VAS			
2-h postoperative	0	0	
4-h postoperative	2.84 ± 0.84	4.80 ± 0.19	< 0.001
6-h postoperative	3.20 ± 0.22	4.91 ± 0.98	< 0.001
24-h postoperative	4.25 ± 0.82	6.06 ± 0.19	< 0.001

Data expressed as frequency (percentage), mean (SD). *P* value was significant if < 0.05

Visual analogue scale (VAS)

The Dexa group demonstrated a significantly longer duration until the first postoperative analgesic request (4.65 ± 1.58 vs. 3.80 ± 1.52 h; P < 0.001) and lower total analgesic consumption of IV ketorolac in the first postoperative 24 h (44.40 ± 22.75 vs. 57.86 ± 18.90 mg; P < 0.001) compared to the control group. additionally, the Dexa group had significantly lower VAS at 4 h postoperative (2.84 ± 0.84 vs. 4.80 ± 0.19; P < 0.001), at 6 h postoperative (3.20 ± 0.22 vs. 4.91 ± 0.98; P < 0.001), and 24 h postoperative (4.25 ± 0.82 vs. 6.06 ± 0.19; P < 0.001). (Table 2)

**Fig. 2:**
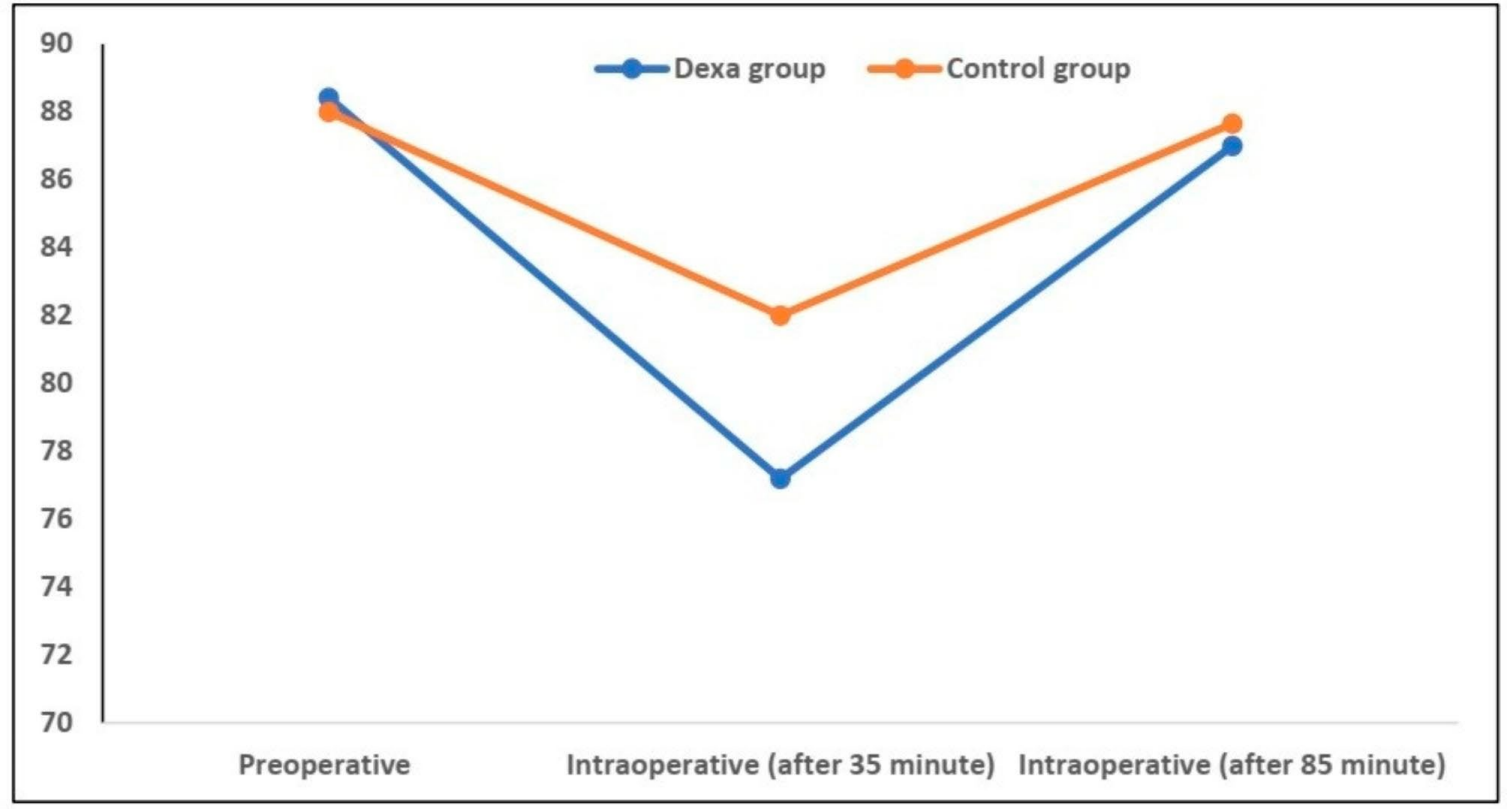
Changes in heart rate among the studied groups, data expressed as mean (SD). P value was significant if  < 0.05. Data were compared by Student t test

**Fig. 3:**
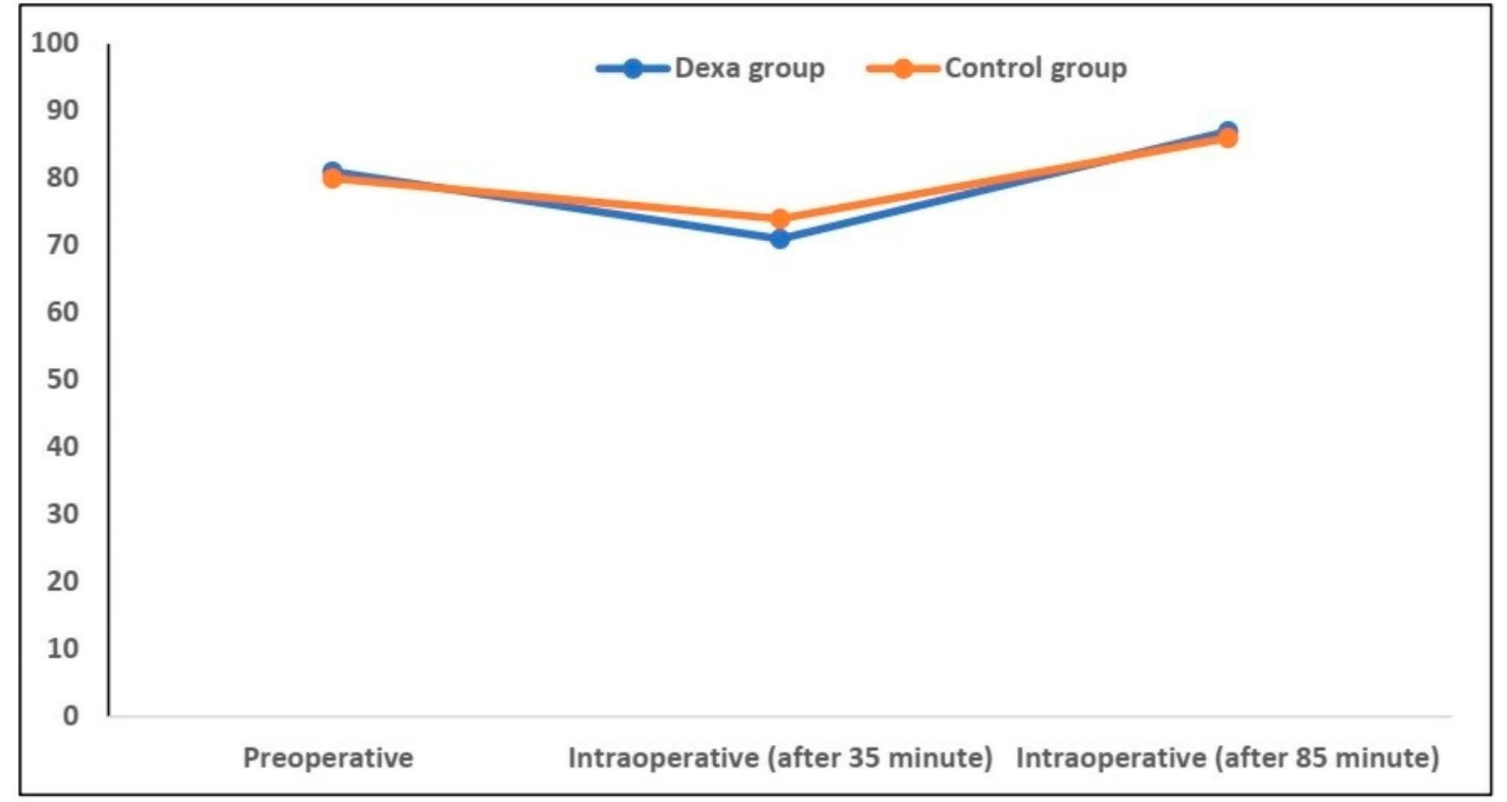
Changes in mean arterial blood pressure among the studied groups, data expressed as mean (SD). P value was significant if  < 0.05. Data were compared by Student t test

There were no significant differences in heart rate and mean arterial blood pressure between both groups, except for a lower intraoperative heart rate after 35 min (77.19 ± 13.62 vs. 82.69 ± 14.86 beats/minute; P < 0.001). However, this difference was not clinically significant. (Figures 2 and 3)

The control group reported a higher incidence of side effects compared to the Dexa group. The Dexa group had significantly lower rates of headache (29% vs. 54%; P < 0.001), nausea (29% vs. 64%; P < 0.001), and vomiting (30% vs. 56%; P < 0.001) compared to the control group (Table 3).

**Table 3:** Reported side effects among the studied groups

	Dexa group (n = 100)	Control group (n = 100)	*P* value*
Headache	29 (29%)	54 (54%)	**< 0.001**
Nausea	29 (29%)	64 (64%)	**< 0.001**
Vomiting	30 (30%)	56 (56%)	**< 0.001**

Data expressed as frequency (percentage). *P* value was significant if < 0.05

*Data were compared by Chi^2^ test

## Discussion

In this study, IV dexamethasone increased the time of two dermatomes regression of sensory level, the total time of sensory and motor block, and the time of the first analgesic request after hyperbaric bupivacaine spinal anesthesia in patients scheduled for lower abdominal surgery.

Our study revealed a significant increase in the duration of two dermatomes regression. However, the difference between the two groups was only 4 min. Additionally, the duration of sensory block differed by 30 min between the two groups. Furthermore, we observed that the difference in the time of the first postoperative analgesic request between the two groups was only one hour.

These findings closely align with the results reported by Juliane Guay et al. [12] who found no significant effects of IV dexamethasone on the duration of 2 dermatomes regression or the total duration of sensory level during spinal anesthesia with isobaric bupivacaine in patients undergoing lower body surgery, although the difference of the concentration of the used bupivacaine in the two studies. It is possible that the timing of dexamethasone administration in this study, immediately prior to intrathecal injection, may have influenced these results. However, it is important to consider that the peak effect of dexamethasone typically occurs between 45 min to one hour after administration [6]. Glucocorticoids altered protein synthesis via gene transcription [14]. As a result, the onset of action of glucocorticoids to suppress the inflammatory mediators is from one to two hours before surgical skin incision [15]. Systemic glucocorticoids analgesic effects have resulted from the inhibition of phospholipase enzyme and accordingly block the cyclooxygenase and the lipoxygenase pathway in the inflammatory chain reaction [15], and it also enhances nociception in inflamed surgical tissue by suppressing the level of bradykinin in the tissues and neuropeptides release from nerve endings [16, 17].

Contrary to these findings, Shalu PS et al., [9] observed that intravenous dexamethasone prolongs the sensory duration and the time of the first postoperative analgesic request after spinal anesthesia with hyperbaric bupivacaine in patients undergoing cesarean section. Although they utilized a similar bupivacaine concentration as in this study, the difference between the two studies could be due to variations in the study populations.

In a study by Kaur H et al., [3] it was found that the addition of intrathecal dexamethasone to hyperbaric bupivacaine resulted in a longer duration of sensory block (311.43 ± 13.59 min) compared to the use of intravenous dexamethasone in this study, intrathecal dexamethasone also provided a similar duration of postoperative analgesia (391 ± 25.51 min) as the IV dexamethasone. Some authors suggest that the analgesic effects of intrathecal corticosteroids are due to their systemic anti-inflammatory effects [18], while others believe that corticosteroids prolong the action of local anesthetics by acting locally on nerve fibers [19]. Further studies are needed to directly compare the effects of IV and intrathecal dexamethasone on sensory duration and the time of first postoperative analgesic request after spinal anesthesia with hyperbaric bupivacaine in the same study.

Although the time to the first request for analgesics was not much prolonged in this study, the dexamethasone group exhibited lower VAS scores and consumed less analgesics compared to the control group. results align with previous studies demonstrating that intravenous dexamethasone provides superior pain relief after surgery [9, 20]. Intermediate doses of dexamethasone, whether administered systemically or perineurally (0.11 to 0.2 mg/kg), have proven to be as higher doses (> 0.2 mg/kg) when used in conjunction with multimodal postoperative analgesia [6, 21, 22]. Furthermore, these intermediate doses have shown to reduce the incidence of postoperative nausea and vomiting without increasing the occurrence of postoperative headache or dizziness [23]. The antiemetic effect of dexamethasone is stems from its central inhibition of prostaglandin synthesis, decreased 5-HT activity, or alternation of blood-brain barrier permeability [24]. Additionally, the anti-inflammatory effect of dexamethasone and its ability to enhance endorphin synthesis in the body may contribute to the prevention of postoperative nausea and vomiting [25]. Consistent with these findings IV dexamethasone in our study significantly reduced the occurrence of postoperative nausea and vomiting.

Limitations of this study:

First, we didn’t assess the effect of dexamethasone on postoperative blood sugar levels or wound healing. However, the existing literature suggests that a single dose of intraoperative dexamethasone does not affect blood sugar, wound healing, or gastrointestinal discomfort [26, 27]. Second, the duration of our study was limited to the first 24 h after surgery, and we did not evaluate any potential effects on patients’ hospital stay or early mobilization. Finally, we administered a single dose of IV dexamethasone immediately before intrathecal injection while the peak effect of dexamethasone is known to occur between 45 min to one hour after administration [6]. Therefore, further larger multicentric studies are needed to determine the optimal timing and dosage of preoperative dexamethasone.

## Conclusion

Although the intravenous administration of dexamethasone had a limited effect on the duration of hyperbaric bupivacaine spinal anesthesia, it improved postoperative VAS scores compared to the control group and decreased overall postoperative analgesic usage. Therefore, it can be considered a valuable addition to postoperative multimodal analgesia strategies, aiming to minimize total analgesic consumption.

## Data Availability

The datasets used and analyzed during the current study are available from the corresponding author upon reasonable request.

## Abbreviations

IV: Intravenous
VAS: Visual Analogue Scale score
ASA: American Society of Anesthesiologists
Dexa group: Dexamethasone group
LAs: Local anesthetics
PACU: Post-anesthesia care unit
